# The Influence of Chronic Cerebral Hypoperfusion on Cognitive Function and Amyloid β Metabolism in APP Overexpressing Mice

**DOI:** 10.1371/journal.pone.0016567

**Published:** 2011-01-27

**Authors:** Mahito Yamada, Masafumi Ihara, Yoko Okamoto, Takakuni Maki, Kazuo Washida, Akihiro Kitamura, Yoshiki Hase, Hidefumi Ito, Keizo Takao, Tsuyoshi Miyakawa, Raj N. Kalaria, Hidekazu Tomimoto, Ryosuke Takahashi

**Affiliations:** 1 Department of Neurology, Graduate School of Medicine, Kyoto University, Kyoto, Japan; 2 Section of Behavior Analysis, Center for Genetic Analysis of Behavior, National Institute for Physiological Sciences, Okazaki, Aichi, Japan; 3 Division of Systems Medical Science, Institute for Comprehensive Medical Science, Fujita Health University, Toyoake, Aichi, Japan; 4 Institute for Ageing and Health, WRC, Campus for Ageing & Vitality, Newcastle University, Newcastle-upon-Tyne, United Kingdom; 5 Department of Neurology, Graduate School of Medicine, Mie University, Tsu, Mie, Japan; National Institute on Aging Intramural Research Program, United States of America

## Abstract

**Background and Purpose:**

Cognitive impairment resulting from cerebrovascular insufficiency has been termed vascular cognitive impairment, and is generally accepted to be distinct from Alzheimer's disease resulting from a neurodegenerative process. However, it is clear that this simple dichotomy may need revision in light of the apparent occurrence of several shared features between Alzheimer's disease and vascular cognitive impairment. Nevertheless, it still remains largely unknown whether the burden of vascular- and Alzheimer-type neuropathology are independent or interdependent. Therefore, we investigated whether chronic cerebral hypoperfusion influences cognitive ability or amyloid β deposition in amyloid precursor protein (APP) overexpressing transgenic mice.

**Methods:**

Two months old mice overexpressing a mutant form of the human APP bearing both the *Swedish* and *Indiana* mutations (APP*_Sw/Ind_*-Tg mice), or their wild-type littermates, were subjected to chronic cerebral hypoperfusion with bilateral common carotid artery stenosis (BCAS) using microcoils or sham operation. Barnes maze test performance and histopathological findings were analyzed at eight months old by 2×2 factorial experimental designs with four groups.

**Results:**

BCAS-operated APP*_Sw/Ind_*-Tg mice showed significantly impaired learning ability compared to the other three groups of mice. Two-way repeated measures analysis of variance showed a synergistic interaction between the APP genotype and BCAS operation in inducing learning impairment. The cognitive performances were significantly correlated with the neuronal densities. BCAS significantly reduced the density of Nissl-stained neurons and silver-stained cored plaques in the hippocampus of APP*_Sw/Ind_*-Tg mice but increased the amount of filter-trap amyloid β in the extracellular-enriched soluble brain fraction, compared to those from sham operated mice.

**Conclusions:**

The results suggest interaction between chronic cerebral hypoperfusion and APP*_Sw/Ind_* overexpression in cognitive decline in mice through enhanced neuronal loss and altered amyloid β metabolism.

## Introduction

Insufficient blood supply to the brain has been shown to lead to cognitive dysfunction [1]. Cognitive impairment resulting from such cerebrovascular insufficiency has been termed ‘vascular cognitive impairment’ (VCI) [1], and is generally accepted to be distinct from Alzheimer's disease (AD) resulting from a neurodegenerative process. However, it is clear that this simple dichotomy may need revision in light of the apparent occurrence of several shared features between AD and VCI. For instance, the two disorders increase in prevalence with age, frequently occur concomitantly, and overlap considerably in their symptomatology, pathophysiology, and comorbidity [2]. Indeed, cerebral hypoperfusion as a result of vascular risk factors such as hypertension, diabetes mellitus, hypercholesterolemia, and smoking is a common vascular component among AD risk factors [3]. Consistent with this, the microvessels in AD neocortex are frequently narrowed, degenerate [4], [5], and amyloid-laiden [6], suggesting a pivotal role of cerebrovascular factors in AD. Furthermore, cerebral hypoperfusion is known to potentiate other deleterious modifiers of AD such as oxidative stress, mitochondrial dysfunction, and neuroinflammation [7], [8]. Thus, neurovascular changes may be key factors in the upstream stage of pathological cascade of AD.

Accordingly, the Nun study has shown that the risk of dementia increases by more than 20 times in AD if the patients exhibit cerebral infarction [9]. The Rush Memory and Aging Project suggested that mixed brain pathologies, mainly comprising of AD pathology and cerebral infarctions, account for most of dementia cases in community-dwelling older persons [10]. Consistent with this, the MRC Cognitive Function and Ageing Study showed attributable risk at death for dementia included small vessel disease (12%), multiple vascular pathologies (9%), and cerebral amyloid angiopathy (7%) in addition to neocortical neuritic plaques (8%) and neurofibrillary tangles (11%) [11]. In this respect, the multifactorial aspects of cognitive impairment may be incorporated into the dynamic polygon hypothesis, which takes into account the contribution of strokes of all sizes, as well as white matter hyperintensities, in parallel to those of plaques and tangles [12].

Nevertheless, it still remains largely unknown whether the burden of vascular- and AD-type neuropathology are independent or interdependent. Elaboration on this point of contention is vital in clarifying the wider question of whether vascular brain injury has additive effects on AD pathogenesis. In this study, we therefore examined whether chronic cerebral hypoperfusion influences cognitive function and amyloid β (Aβ) neuropathology in APP overexpressing mice [8], [13], [14], [15]. This may determine whether the burden of vascular- and AD-type neuropathology is interdependent in the development of dementia syndrome, and may provide evidence linking chronic hypoperfusion with neurodegeneration.

## Materials and Methods

### Animals, treatments, and surgical procedures

We used human APP-Tg mice J20 overexpressing the familial AD-linked mutation carrying a mutant form of the human APP bearing the both *Swedish* (K670N/M671L) and the *Indiana* (V717F) mutations (APP*SwInd*) [16], imported from the Jackson Laboratory (USA). This J20 high expressing transgenic line is different from the J9 low expressing line used in our earlier work [5], [17]. Mice were screened for transgene expression by PCR, and heterozygous mice were mated with nontransgenic C57BL/6J mice. All male heterozygous transgenic mice were given access to food and water *ad libitum*. At 2 months of age, mice of heterozygosity and their non-Tg littermates were subjected to either sham operation or bilateral common carotid artery stenosis (BCAS) using microcoils (four groups) [8], [13], [14], [15], [17]. Under halothane anesthesia (2%), the common carotid arteries were exposed through a midline cervical incision, and a microcoil with a diameter of 0.18 mm was applied to the bilateral common carotid artery (while maintaining the rectal temperature between 36.5 and 37.5°C). Those in the control group were sham-operated, which involved bilateral exposure of the common carotid arteries only. Body weight, rectal temperature, and blood pressure of tail artery was monitored in BCAS- and sham-operated mice. After the operation, the mice were housed in cages with a 12-hour light/dark cycle (lights on at 7:00 AM) with access to food and water *ad libitum*. At 6 months post-BCAS, the mice were tested for altered behavior. This study was carried out in strict accordance with the guidelines for animal experimentation from the Animal Research Committee, Kyoto University. The protocol was approved by the Animal Research Committee, Kyoto University (Permit Number: MedKyo08526). The raw data of the behavioral tests have been disclosed in the mouse behavioral phenotype database (http://www.mouse-phenotype.org/).

### Behavioral tests

#### Neurological screen

Neurological screening was conducted in 8-month-old male mice (6 months after operation), as previously described [8], [18], [19]. Ear twitch, whisker touch, and righting reflexes were also evaluated.

#### Barnes maze test for reference memory

The Barnes maze test was performed in the 8-month-old male mice (6 months post-operation). The task was conducted on ‘dry land’, a white circular surface, 1.0 m in diameter, with 12 holes equally spaced around the perimeter (O' Hara & Co., Tokyo, Japan) [20], [21]. The circular open field was elevated 75 cm from the floor. A black Plexiglas escape box (17×13×7 cm) containing paper cage bedding on its floor was located under one of the holes. The hole above the escape box represented the target, analogous to the hidden platform in the Morris task. The location of the target was consistent for a given mouse but was randomized across mice. The maze was rotated daily, with the spatial location of the target unchanged with respect to the visual room cues, in order to prevent bias based on olfactory or proximal cues within the maze. The first training session was started when wild type mice and BCAS-operated mice were 8-months-old (6 months after the BCAS operation). One trial per day for 7 successive days and two trials per day for next 6 successive days were conducted except for no trial on day 6 and one trial on the last day. A probe trial was conducted 24 hours after the last training session without the escape box in order to confirm that this spatial task was performed based on navigation using distal environment room cues. Time of latency to reach the target hole, number of errors, distance to reach the target hole, and time spent around each hole were recorded by video tracking software (see ‘Image Analysis’). To assess long-term retention, a second probe trial was applied a week after probe test 1 and additional one session of retraining.

#### Image analysis

The applications used for the behavioral studies were based on the National Institutes of Health's Image program (available at http://rsb.info.nih.gov/nih-image/) and ImageJ program http://rsb.info.nih.gov/ij/, which were modified for each test by Tsuyoshi Miyakawa (available from O'hara & Co., Tokyo, Japan).

### Histological investigation

#### Tinctorial and immunohistochemical staining

Mice were deeply anesthetized with sodium pentobarbital and were perfused transcardially with 0.01 mol/L phosphate-buffered saline (PBS) and then with a fixative containing 4% paraformaldehyde (PFA) and 0.2% picric acid in 0.1 mol/L phosphate buffer (PB, pH 7.4). The brains were post-fixed in 4% PFA in 0.1 mol/L PB, and were stored in 20% sucrose in 0.1 mol/L PB (pH 7.4). Posterior (hippocampal) portion of each brain was dissected in consultation with a brain map [22]. The coordinates from the bregma measured from –1.8 to –2.2 mm for histochemical analyses. The posterior portion was embedded in paraffin and sliced into 6 µm-thick coronal sections and then subjected to hematoxylin and eosin (H&E) staining and modified Bielschowsky staining. For immunohistochemistry, serial sections cut on a microtome were incubated overnight at 4°C with anti-amyloid beta 42 (Aβ42; Calbiochem; diluted 1∶500) after retrieval by formic acid for 2 minutes and 0.05% trypsin for 15 minutes. The sections were subsequently treated with the appropriate biotinylated secondary antibodies (diluted 1∶200; Vector Laboratories, Burlingame, CA) and visualized with 0.01% diaminobenzidine tetrahydrochloride and 0.005% H_2_O_2_ in 50 mmol/L Tris HCl (pH 7.6).

#### Measurement of neuronal density

The neuronal density in the cerebral cortex and the hippocampus was counted blindly by a second investigator (Y. Okamoto). Nissl-stained coronal sections of the brain (–1.8 to –2.2 mm from the bregma) obtained from the four groups (n = 4–7) were used for counting the neurons by setting regions of interest in the cerebral cortex (the watershed area of anterior and middle cerebral artery) and the hippocampal CA1 and CA3 regions.

#### Measurement of cored plaque density

The density of cored plaques was counted blindly by a second investigator (Y. Okamoto) in the cortex and the whole hippocampus. The density of cored plaques per unit area was calculated by dividing the number of cored plaques by the number of whole pixels of each captured image.

#### Measurement of Aβ42-stained area

Aβ42-stained sections were captured by using digital microscope system, BZ-9000 (Keyence, Osaka, Japan). A unified threshold value (155/255) was used to binarize the images. Captured images were separated into the cortex, the hippocampus and the other area by the Photoshop 7.0 (Adobe Systems Inc.). Image analysis was performed by ImageJ 1.40 g software (NIH). The number of Aβ42-positive pixels was divided by the entire pixels to yield %Aβ42-stained area. The person performing the analysis was blinded to the animal groups.

### Protein extraction and filter-trap assay

The protein samples for the filter assay were extracted according to the method proposed by Lesne et al [23], with some modification. Briefly, hemi-forebrains were harvested in Tris-buffered saline (pH 7.6) and a protease inhibitor cocktail (Sigma, USA). Soluble, extracellular-enriched proteins were collected from homogenized lysates following centrifugation for 40 min at 100,000×g. The protein concentration of the samples was measured according to the Bradford method and equal amount of protein was subjected to filter assay.

The extracellular-enriched proteins from the brains of four pairs of sham-operated or BCAS-treated littermates were subjected to vacuum filtration through a 96-well dot blot apparatus (Bio-Rad Laboratories, USA) containing 20 nm pore-sized nitrocellulose membranes. The resultant membranes were then incubated with primary antibody (6E10; diluted 1∶1000) at 4°C overnight. The membranes were then blocked by TBS containing 4% skim milk, and incubated with HRP-linked anti-mouse IgG secondary antibody (Mouse TrueBlot ULTRA, eBioscience; diluted 1∶1000) for 1 h. The membranes were developed with the ECL plus Western Blotting Analysis System (GE Healthcare). The membrane was digitally captured with LAS-3000 (Fuji, Tokyo, Japan). The LAS-3000 imaging system has a CCD camera which is equipped with shading correction without automatic gain control, and has an ample and linear dynamic range (from 0 to 4.0 OD values). Images were processed using Multi Gauge v3.1 software (Fujifilm). The intensity of each spot was determined by measuring the AUC-BG/mm^2^ (AUC: area under the curve, BG: background).

### Statistical analysis

Statistical analysis was conducted using StatView (SAS Institute). Data were analyzed by two-way repeated measures analysis of variance (ANOVA) or one-tailed t-test, unless otherwise noted. A one-tailed t-test was used for two-group comparison based on the clear directional prediction. Values in the graphs are expressed as mean±SEM. p<0.05 was considered statistically significant.

## Results

### General characteristics

#### Physical changes, sensory motor reflexes, and nociception

The surgical procedures for BCAS were accomplished within 15 minutes on each mouse. By day 10 post-BCAS, less than 10% of the mice had died. The APP genotype did not affect the survival rate. There were no significant differences among the four groups of mice in terms of their physical characteristics such as body weight, temperature and blood pressure. Mean body weight in non-Tg/sham, non-Tg/BCAS, APP*_Sw/Ind_*-Tg/sham, and APP*_Sw/Ind_*-Tg/BCAS mice was 25.8 g, 26.5 g, 24.5 g, and 23.7 g at the operation (2-month-old), respectively, with no significant differences among the four groups. There were also no significant differences in sensory-motor reflexes (percent with quick response of ear twitch, normal response of whisker twitch and acoustic startle response) and physical strength (grip strength and wire hang).

### Behavioral tests

#### Reference memory test

**Figure 1:**
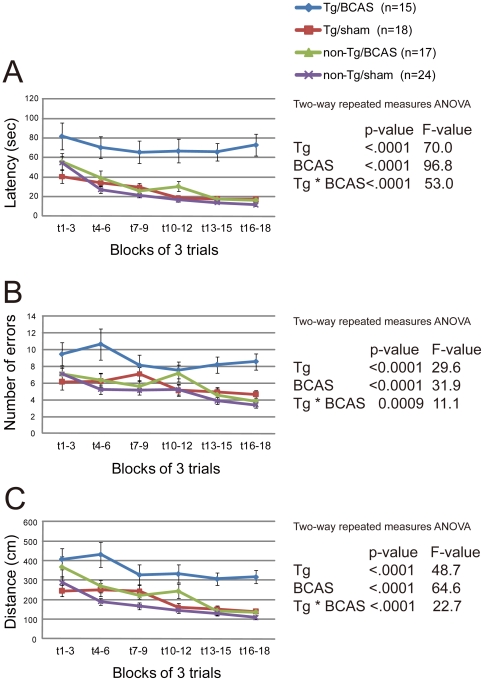
Interaction between APP overexpression and chronic cerebral hypoperfusion. Barnes maze test showed that APP*_Sw/Ind_*-Tg/BCAS mice exhibited prolonged latency to reach the desired target (A), increased number of errors (B), and increased distance before reaching the desired target (C) compared to the other groups.

The Barnes maze test was performed to investigate reference memory at 6 months post-BCAS (8-month-old). There were significant differences between the APP*_Sw/Ind_*-Tg/BCAS mice and the other three groups of mice; the APP*_Sw/Ind_*-Tg/BCAS mice exhibited prolonged latency to reach the desired target (Figure 1A), increased number of errors (Figure 1B), and increased distance in reaching the desired target (Figure 1C) compared to the other groups of mice. These results suggest that the learning of the APP*_Sw/Ind_*-Tg/BCAS mice was significantly impaired. In two-way repeated measures ANOVA, there was a synergistic interaction between genotype and BCAS. The mutant APP gene and BCAS synergistically worsened learning ability (Figure 1A–C).

In the probe trial conducted 24 hours after the last training session, the time spent around the target hole by the APP*_Sw/Ind_*-Tg/BCAS mice was significantly shortened (p = 0.0083) compared to the non-Tg/BCAS mice (p = 0.6881). There was no interaction between the mutant APP genotype and BCAS operation (p = 0.540). To assess the long-term retention of spatial memory, a second probe test was conducted 7 days after the last training trial. Both the mutant APP genotype and BCAS operation shortened the time spent around target hole (APP, p = 0.0104; BCAS, p = 0.0202), but there was no interaction between genotypes and surgery conditions (p = 0.2617).

### Histological findings

#### Neuronal density

**Figure 2:**
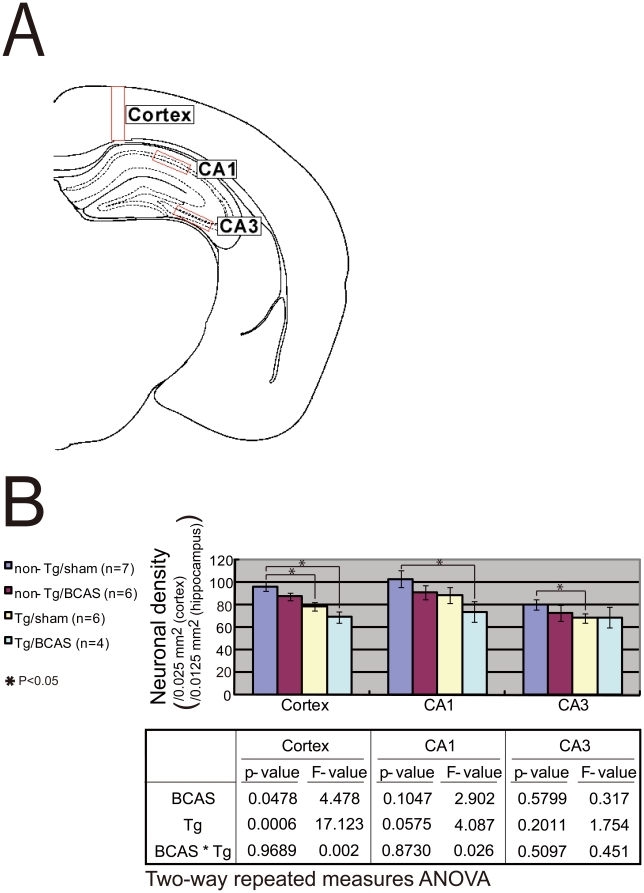
Neuronal density in the cerebral cortex and the hippocampal CA1 and CA3 areas. (A) The schematic illustration of the regions of interest depicted in the cerebral cortex (size, 0.05×0.5 mm) and the hippocampal CA1 and CA3 areas (0.05×0.25 mm each) of the coronal section of the brain. (B) The density of Nissl-stained neurons was lowest in APP*_Sw/Ind_*-Tg/BCAS mice.

The density of Nissl-stained neurons was reduced in the APP*_Sw/Ind_*-Tg/BCAS mice with increment in the following order: non-Tg/sham > non-Tg/BCAS > APP*_Sw/Ind_*-Tg/sham > APP*_Sw/Ind_*-Tg/BCAS mice in the cerebral cortex and hippocampal CA1 and CA3 (Figure 2). There were significant differences in neuronal density between non-Tg/sham and APP*_Sw/Ind_*-Tg/BCAS or APP*_Sw/Ind_*-Tg/sham mice in the cerebral cortex, between non-Tg/sham and APP*_Sw/Ind_*-Tg/BCAS mice in the hippocampal CA1 region, and between non-Tg/sham and APP*_Sw/Ind_*-Tg/sham mice in the hippocampal CA3 region. There was no interaction in neuronal density between mutant APP overexpression and BCAS in the three areas (cortex, p = 0.969; CA1, p = 0.873; CA3, p = 0.510). Mutant APP overexpression and BCAS seemed to have additive effects on the neuronal density in the cerebral cortex and CA1 region.

The neuronal densities in the cerebral cortex and hippocampal CA1 and CA3 were significantly and inversely correlated with the cognitive performances on the Barnes maze test, such as time of latency to reach the target hole, number of errors and distance to reach the target hole (trials 16–18) (Table 1).

**Table 1:** Significant inverse correlations of the cognitive performances with the neuronal densities.

	Cortex	CA1	CA3
Latency	r = −0.458 (p<0.0001)	r = −0.291 (p = 0.0149)	r = −0.290 (p = 0.0152)
Number of errors	r = −0.379 (p = 0.0012)	r = −0.301 (p = 0.0116)	r = −0.319 (p = 0.0072)
Distance	r = −0.417 (p = 0.0003)	r = −0.314 (p = 0.0083)	r = −0.272 (p = 0.0237)

r = correlation coefficient.

#### Modified Bielschowsky pathology

**Figure 3:**
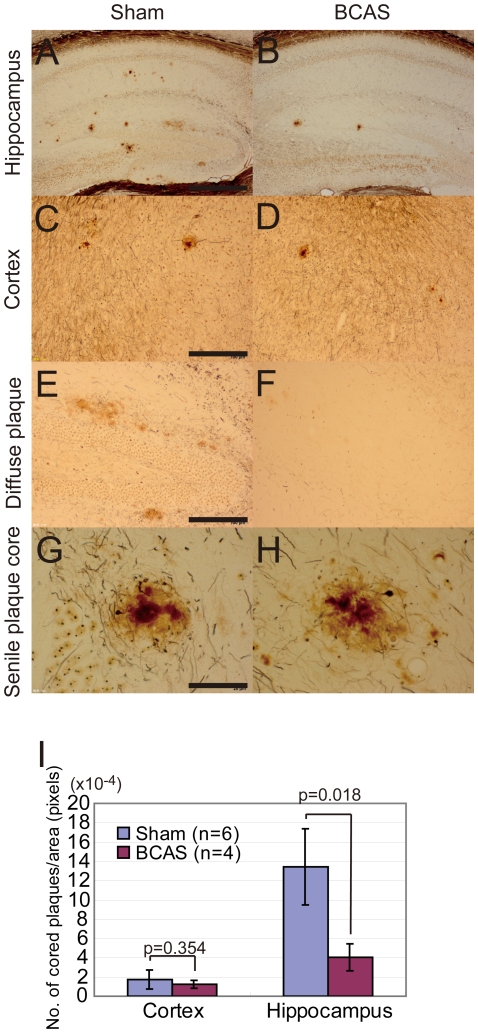
Chronic cerebral hypoperfusion affected the number of silver-stained cored plaques in the APP*_Sw/Ind_*-Tg mice. Modified Bielschowsky staining showed cored plaques in the hippocampus (A, B, G, H) and the cerebral cortex (C, D), and diffuse plaques in the hippocampus (E, F) of the APP*_Sw/Ind_*-Tg/sham mice (n = 6; A, C, E, G) and APP*_Sw/Ind_*-Tg/BCAS mice (n = 4; B, D, F, H). Number of cored plaques was counted in the cortex and the hippocampus (I). Scale bars: 400 µm (A, B), 300 µm (C-F), 80 µm (G, H).

In non-Tg mice, there was no amyloid pathology in the brains of the BCAS or sham operated mice. The analyses subsequently involved comparing APP*_Sw/Ind_*-Tg/sham and APP*_Sw/Ind_*-Tg/BCAS mice. Modified Bielschowsky staining showed that the number of silver-stained cored plaques was lower in the APP*_Sw/Ind_*-Tg/BCAS mice compared to the APP*_Sw/Ind_*-Tg/sham mice (compare Figure 3A and 3B, and Figure 3C and 3D). The difference was significant in the hippocampus (p = 0.018) but not in the cerebral cortex (p = 0.354) (Figure 3I). Diffuse plaques were numerous in the APP*_Sw/Ind_*-Tg/sham mice but fewer in the APP*_Sw/Ind_*-Tg/BCAS mice (Figure 3E and 3F). The marked difference in diffuse plaques was clearly evident without further need for quantification. By contrast, cored plaques (senile plaque cores) were not morphologically different between the two groups of mice (Figure 3G and 3H).

#### Aβ1-42 pathology

**Figure 4:**
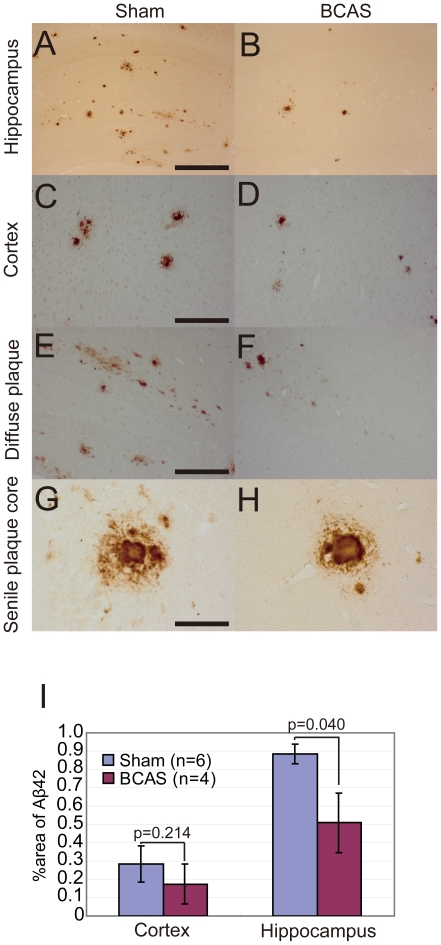
Chronic cerebral hypoperfusion reduced the Aβ1-42 stained area in the APP*_Sw/Ind_*-Tg mice. Immunohistochemistry for Aβ1-42 showed intense staining in the hippocampus (A, B, E, F) and in the cerebral cortex (C, D) of the APP*_Sw/Ind_*-Tg/sham mice (n = 4; A, C, E, G) and APP*_Sw/Ind_*-Tg/BCAS mice (n = 6; B, D, F, H). (I) The area positive for Aβ1-42 was analysed in the cortex and the hippocampus. Scale bars: 400 µm (A, B), 300 µm (C–F), 80 µm (G, H).

The Aβ1-42 immunostaining showed similar findings to modified Bielschowsky staining. The number of Aβ-positive plaques was lower in the APP*_Sw/Ind_*-Tg/BCAS mice compared to the APP*_Sw/Ind_*-Tg/sham mice (compare Figure 4A and 4B, and Figure 4C and 4D). The %Aβ42-stained area showed a decrement in the APP*_Sw/Ind_*-Tg/BCAS mice compared to the APP*_Sw/Ind_*-Tg/sham mice in both the cortex and hippocampus. The difference reached significance in the hippocampus (Figure 4I). However, in contrast to modified Bielschowsky staining, Aβ immunostaining showed intense positivity for both diffuse plaque (Figure 4E and 4F) and cored plaque (Figure 4G and 4H). The Aβ-positive diffuse plaques was less frequently observed in the APP*_Sw/Ind_*-Tg/BCAS mice compared to the APP*_Sw/Ind_*-Tg/sham mice, while cored plaques showed no morphological differences between the two groups of mice (Figure 4G and 4H).

### Biochemical findings

**Figure 5:**
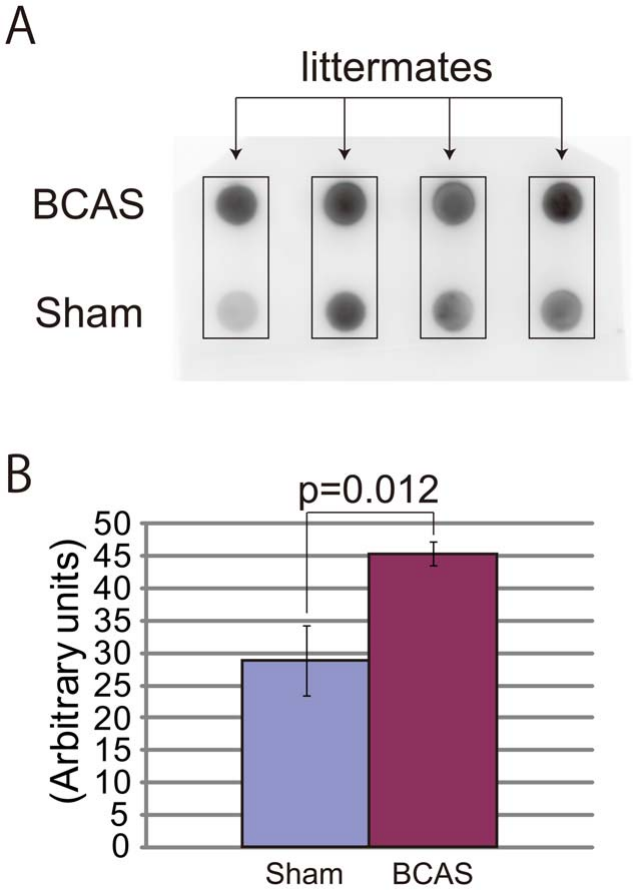
Chronic cerebral hypoperfusion increased filter-trap Aβ in the extracellular-enriched, soluble brain fraction. (A) Filter-trap, Aβ-immunoreactive spot density was increased in samples from BCAS-treated mice, compared to sham-operated littermates. (B) Spot density was significantly increased in BCAS-treated mice (paired t-test; p = 0.0231, n = 4).

The filter-trap assay showed that BCAS-treated mice had significantly higher levels of filter-trap Aβ (p = 0.012) in the extracellular-enriched, soluble brain fraction, as shown in the four littermate pairs (Figure 5).

## Discussion

This behavioral, histological, and biochemical study demonstrates that chronic cerebral hypoperfusion accelerates reference memory impairment in conjunction with hippocampal neuronal loss in the brain of APP*_Sw/Ind_* mice. Chronic cerebral hypoperfusion (6 months) or APP*_Sw/Ind_* overexpression impaired reference memory in mice [16], but a novel finding presented here is that chronic cerebral hypoperfusion and APP*_Sw/Ind_* overexpression interdependently disrupted reference memory (Figure 1). Although a threshold for behavioral deficits may have been crossed when a certain number of hippocampal neurons are lost (threshold effect), the results suggest that burden of vascular- and AD-type lesions interdependently contribute to the development of components of the dementia syndrome, and strengthen the notion that vascular risk factors, if present, should be thoroughly controlled in clinically probable AD patients [24]. The vascular-type lesions reproduced in the BCAS model are oligemic e.g. non-infarctional indicating that chronic hypoperfusion may accelerate AD neuropathology in a latent manner over an extended period of time via enhanced neuronal loss and altered Aβ metabolism. Although we did not focus on aging aspects in particular, this effect is likely to be more pronounced in older animals [4].

Several studies have reported that chronic ischemia/hypoxia mechanistically contribute to AD pathogenesis via alteration of Aβ metabolism. In *Swedish* mutant APP transgenic mice (APP23), long-term hypoxia has been shown to markedly increase Aβ deposition and neuritic plaque formation and potentiate the memory deficit by increasing β-site APP cleaving enzyme 1 (BACE1) gene transcription and expression, primarily mediated by the binding of hypoxia-inducible factor-1α to the BACE1 promoter [25], [26]. BACE1 activation and resultant Aβ40 overproduction has also been reported in Tg2576 mice following energy insufficiency by pharmacological agents (insulin, 2-deoxyglucose, 3-nitropropionic acid, or kainic acid) [27]. Such findings collectively suggest that the energy/oxygen deficiency facilitates AD pathogenesis by BACE1 elevation and Aβ overproduction.

In this study, the degree of Aβ deposition and cored plaque formation was suppressed following chronic cerebral hypoperfusion and APP*_Sw/Ind_* overexpression, although this interaction resulted in the augmentation of reference memory impairment and hippocampal neuronal loss. This group has previously reported that chronic cerebral hypoperfusion increased the level of aggregated Aβ of no less than 200 nm in diameter in the soluble extracellular-enriched brain fraction of a relatively low-expressor line (J9) of APP*_Sw/Ind_* mouse [17]. The current study, which used a high-expressor line (J20) of APP*_Sw/Ind_* mouse, also demonstrated that the amount of filter-trap Aβ in the extracellular-enriched, soluble fraction significantly increased following 6 months of cerebral hypoperfusion. By contrast, insoluble Aβ species that cause senile plaques were reduced in this J20 line after cerebral hypoperfusion but had not been analysed in the J9 line due to their scarcity in the brain [17]. Therefore, soluble, but not insoluble, Aβ species may play a direct role in neurotoxicity or neuronal loss and resultant behavioral abnormalities in the hypoperfused APP*_Sw/Ind_* mice. Insoluble plaque cores are known to be largely inactive but sequester synaptotoxic Aβ dimers [28]. Insoluble Aβ deposition may be disassembled by the inflammation caused by cerebral hypoperfusion as ischemia is known to activate macrophages and reduce senile plaques [29]. Alternatively, chronic cerebral hypoperfusion may interrupt Aβ aggregation process, causing a shift in Aβ solubility. Thus, this study suggests that insoluble Aβ1–42 may not be the causative factor, but rather a resultant epiphenomenon, in cognitive deterioration in the APP*Sw/Ind* mice. These findings are consistent with a recent report that found Aβ oligomer-induced pathology in the absence of amyloid plaques in APP-Tg mice expressing the E693Delta mutation [30]. They also concur with the findings of several earlier studies that found environmental enrichment consistently prevented cognitive decline in AD model mice, but changed the amount of senile plaques variably, according to the experimental conditions (the senile plaques increased [31], decreased [32], or remained the same [33] despite the consistent cognitive improvement).

Ischemia increases the vulnerability of neurons to Aβ peptide by impairing calcium regulation [34]. Cellular calcium homeostasis is also disrupted by AD-causing mutations in presenilin-1 and APP in cultured neurons and transgenic mice [35]. Therefore, the synergistic interaction between ischemia and neurodegeneration may be mediated by calcium dysregulation. In accordance with this hypothesis, presenilin-1 mutant knock-in mice exhibit greater severity of brain injury and poorer behavioural outcome after focal cerebral ischemia/reperfusion [36]; moreover, neuronal elevation of intracellular calcium levels is enhanced after glucose deprivation and chemical hypoxia in cortical cell cultures from the same animals [36]. Thus, disturbance in calcium homeostasis may provide a mechanistic link between ischemia and neurodegeneration, and may underlie the observed synergistic interaction between APP overexpression and hypoperfusion in cognitive impairment induction.

The cognitive decline arising from human AD or VCI is known to progress over a period of decades. A report using 3xTg-AD mice (∼3 months of age) and APP*_Sw/Ind_* mice (16–17 months) indicated that blood flow insufficiency should be prolonged enough to accelerate AD pathology; here, the use of captopril (a commonly used anti-hypertensive drug) and resultant modest inhibition (∼30%) of brain angiotensin-converting enzyme (Aβ-degrading) activity for 28 days did not affect Aβ catabolism [37]. This suggests that cerebral autoregulatory capacity and redundant Aβ-degrading pathways counteract Aβ overproduction triggered by blood flow insufficiency at least for a short period of time. Consistent with this, the authors have previously shown anatomical and metabolic abnormalities in the hippocampus after 6 months of cerebral hypoperfusion [8]. It is therefore apparent that cerebral hypoperfusion over a prolonged period of time (probably encompassing a significant proportion of life) is necessary to replicate the condition found in humans.

A limitation of this study is the lack of information on the temporal profile of cerebral blood flow (CBF) in our cohort of the APP*_Sw/Ind_* mice. This group has previously monitored the CBF of wild-type C57BL/6J mice immediately after the BCAS operation up to 3 months post-BCAS, indicating that the CBF temporarily decreased to 60 to 70% of the control value but gradually recovered to a level of >80% at 1 to 3 months [8]. Given the cerebrovascular dysfunction in the J20 line of the APP*_Sw/Ind_* mice [38], the CBF may be further jeopardized in the APP*_Sw/Ind_* mice compared to C57BL/6J mice after BCAS operation. However, CBF measurement procedures requiring anesthesia after the behavioral study were avoided in order to minimize the influence of anesthesia on the correlation analysis between cognitive performance and histopathological findings. Therefore, future study should investigate whether APP overexpression accelerates CBF reduction in the BCAS brain.

Another limitation is that Aβ was not measured in a quantitative fashion and plaque load or neuronal cell counts were determined without using stereological approaches. However, two different histological methods (modified Bielschowsky staining and Aβ1-42 immunostaining) showed the similar trend of decreasing plaque load after cerebral hypoperfusion in both the cortex and hippocampus, making a chance finding less likely. Sampling of each mouse using stereological methods will further delineate our findings in a future study.

In conclusion, this study showed that chronic cerebral hypoperfusion accelerated reference memory impairment and hippocampal neuronal loss together with reduced Aβ deposition and cored plaque formation but an increased amount of filter-trap Aβ in the extracellular-enriched soluble brain fraction in APP*_Sw/Ind_* mice. This suggests interaction between chronic cerebral hypoperfusion and APP overexpression for cognitive decline through altered Aβ metabolism.
